# Cerebrospinal Fluid α-Calcitonin Gene-Related Peptide: A Comparison between Alzheimer’s Disease and Multiple Sclerosis

**DOI:** 10.3390/biom12020199

**Published:** 2022-01-24

**Authors:** Giulio Papiri, Arianna Vignini, Luigi Capriotti, Paola Verdenelli, Sonila Alia, Alice Di Paolo, Chiara Fiori, Sara Baldinelli, Mauro Silvestrini, Simona Luzzi

**Affiliations:** 1 Neurology Clinic, Azienda Ospedaliero Universitaria, Ospedali Riuniti di Ancona, 60126 Torrette di Ancona, Italy; giulio.papiri@outlook.it (G.P.); chiarafiori@live.it (C.F.); sara.baldinelli@ospedaliriuniti.marche.it (S.B.); m.silvestrini@univpm.it (M.S.); s.luzzi@univpm.it (S.L.); 2 Section of Biochemistry, Department of Clinical Sciences, Biology and Physics, Università Politecnica delle Marche, 60126 Ancona, Italy; s.alia@pm.univpm.it (S.A.); a.dipaolo@pm.univpm.it (A.D.P.); 3 Anesthesiology and Intensive Care Unit, Ospedale Provinciale “Madonna del Soccorso”, 63074 San Benedetto del Tronto, Italy; luigi.capriotti@sanita.marche.it (L.C.); paola.verdenelli@sanita.marche.it (P.V.)

**Keywords:** α-CGRP, Alzheimer’s disease, multiple sclerosis, neuropsychology, biomarkers, cognitive impairment

## Abstract

Alzheimer’s disease (AD) and Multiple Sclerosis (MS) represent an emerging health problem on a global scale, as they are responsible for a significant contribution to the burden of disability in Western countries. Limited numbers of cerebrospinal fluid (CSF) diagnostic markers are available for each disease (amyloid and tau deposition markers for AD and oligoclonal bands for MS) representing mostly state markers that provide few, if any, clues about the severity of the clinical phenotype. α-CGRP is a neuropeptide implied in nociception, vasodilation, synaptic plasticity and immune functions. This neuropeptide is expressed in encephalic regions connected to memory, attention, autonomic and behavioral functions and is also expressed by spinal motor neurons. The present work confronted α-CGRP levels between 19 AD, 27 MS and 17 control subjects using an ELISA/EIA assay. We measured higher CSF α-CGRP contents in control subjects with respect to AD, as shown in previous studies, as well as in MS patients in comparison to AD. The control subjects and MS patients did not significantly differ between each other. We did not observe a relationship between CSF protein content, albumin quotient and α-CGRP. We also describe, retrospectively, an association between higher CSF CGRP content and higher MRI overall lesion count in MS and between lower α-CGRP and worse attention and visuo-perceptual skills in AD. We speculate that α-CGRP could be differentially involved in both disabling diseases.

## 1. Introduction

Alzheimer’s disease (AD) represents the most common cause of degenerative dementia. It is characterized by progressive impairment of cognition, beginning in most cases with short term memory impairment, which is followed over time by the involvement of other cognitive domains, mainly attention and executive functions. It is estimated that 50 million people worldwide will be affected with Alzheimer’s disease in the next decades [1]. While the etiology remains elusive, its complex pathogenesis involves progressive neurochemical imbalances thought to begin from Amyloid Beta and Hyperphosphorylated Tau aggregation and spreading [2]. 

Many studies have found evidence regarding the activity within the whole pathogenetic framework, even from early phases, of inflammatory cascades, in both animal models and human studies, through cerebrospinal fluid (CSF) analyses [3,4]. Multiple Sclerosis, on the other hand, represents the most common demyelinating disease of the Central Nervous System (CNS). Its etiology, currently unknown, is thought to be multifactorial [5]. It is considered to be an autoimmune disorder that affects the CNS by inducing recurrent or progressive demyelination resulting in white and gray matter lesions that accumulate over time, thus, producing progressive neurological disability. 

MS may show, apart from strictly inflammatory mechanisms, processes in common with neurodegenerative diseases, such as synaptic and mitochondrial dysfunction [6,7]. Some studies suggested that Amyloid Beta is also related to white matter damage in AD, while also influencing remyelination processes in MS [8]. Furthermore, in MS, cognitive impairment is consistently recognized as a common event, beginning from the early phases. Both AD and MS represent a significant source of disability in western countries, albeit in different age ranges [9]. 

Even though the therapeutic approaches differ significantly between the two conditions, early diagnosis remains crucial in both. There is, therefore, a significant need for mechanistic biomarkers. For AD, the better characterized markers are those of amyloid and tau deposition, which allow linking the presence of cognitive impairment to specific proteinopathies as hallmarks of the disease [10]. As for MS, the best characterized laboratory marker is represented by CSF oligoclonal bands, which represent a proxy for dissemination in time [11].

Currently, for both diseases, there are no CSF markers able to predict distinct clinical phenotypes, such as impairment in specific cognitive domains in AD or a relapsing-remitting vs. progressive course in MS. Moreover, there are no known markers related to the degeneration of specific anatomic systems, such as the temporo-mesial networks in AD or cerebral cortex and spinal cord in MS.

Alfa-Calcitonin Gene Related Peptide (α-CGRP) is a 37-aa neuropeptide expressed ubiquitously from alternative splicing of the CALCA gene mostly in the peripheral nervous system and CNS [12]. It has been regarded, since its discovery, to play a prominent role in vasodilation, nociception, neurogenic inflammation and interoception. It possesses several functions in nearly every organ and system. In fact, it serves as a co-transmitter in both the peripheral nervous system (PNS) and CNS. Its role is considered pivotal in the processes implied in the pathophysiology of migraines [13]. 

In the PNS, it is mainly expressed by sensory ganglia, while in the CNS, it shows a widespread expression, predominant in subcortical structures, such as parabrachial nucleus, ventral tegmental area, bed nucleus of stria terminalis, amygdala and cerebellum [14]. In the spinal cord, it is mainly produced by motor neurons, where it is stored and released with acetylcholine [15]. 

Its central functions have been characterized less extensively. Many animal studies have shown that it profoundly affects behavior [16], as well as biological processes related to mood and cognition, such as hippocampal neurogenesis and synaptic plasticity [17]. In disease scenarios, it is thought to be released by neurons in response to various stressful stimuli [18]. Its properties have been studies most extensively in models of depression, where it has been defined as a possible disease trait marker [16,19,20]. 

In addition, it was shown to exert profound regulatory effects on the immune system, with potential both anti-inflammatory and pro-inflammatory properties [18,21]. Many studies have shown neuroprotection in different contexts, including ischemia-reperfusion injury and experimental allergic encephalomyelitis (EAE) [22,23,24]. As for human studies, there are currently no data about α-CGRP in CSF in MS patients. As for dementia, Mathé and colleagues, in 2002, presented a significant reduction in α-CGRP CSF content in patients affected by degenerative non-vascular dementia (AD/FTD) with respect to age matched controls [25]. They also observed a preservation of the Calcitonin/α-CGRP ratio in dementia, arguing that their finding might be expression of generalized atrophy. 

Other studies have shown that α-CGRP CSF concentration is relatively independent from sex, age and medication [26]. It was observed that α-CGRP content might show a positive relationship to protein content when the latter is pathologically increased, such as in infectious disorders, where the blood brain barrier is damaged [27]. 

Na and colleagues, in a recent study, observed that α-CGRP might be involved in the pathogenesis of AD, from the early phases, in 5XFAD mice, since treatment with α-CGRP receptor blockers yielded amelioration of the disease phenotype [28]. Thus, considering evidence from preclinical studies linking α-CGRP to both neuronal survival and immune homeostasis, given the coexistence in both diseases of an inflammatory and degenerative component, it might be considered as a possible biomarker in both scenarios. 

Given the relative lack of knowledge regarding α-CGRP CSF concentration and dynamics in MS, the present study aimed at: (i) measuring and confronting α-CGRP CSF concentration in AD, MS patients and control subjects by using ELISA/EIA methods, as previous studies have always been carried out using the RIA method; and (ii) studying the relationship between α-CGRP CSF concentration and clinical features in both diseases, such as white matter lesion burden and cognitive performance in specific neuropsychological domains. Lastly, we assessed whether the α-CGRP concentration and CSF physical–chemical characteristics relate differently in each disease.

## 2. Materials and Methods

### 2.1. Patients

#### 2.1.1. Control Subjects

We enrolled 17 control subjects in the study. The control cohort was composed by patients referring to the Orthopaedics Unit of the “Madonna del Soccorso” Hospital (San Benedetto del Tronto, Italy) for either elective Hip/Knee arthroplasty or Anterior Cruciate Ligament repair surgery in spinal anesthesia. This study was carried out in accordance with the principles of Declaration of Helsinki and was approved by the Marche Polytechnic University Review Board (ID 1922, CERM 2021 12).

Subjects whose medical history was negative for neurologic diseases (neurodegenerative disorders, symptomatic ischemic stroke or brain hemorrhage, recent brain infection of any cause, history of encephalitis, chronic or episodic migraine), psychiatric comorbidities (major depression, generalized anxiety disorder or psychosis according to the DSM-IV classification), active oncologic disease, autoimmune disease, uncompensated diabetes or thyroid disorders, recent heart attack or advanced heart failure (NYHA class III or IV), respiratory failure or chronic lung diseases were enrolled in the study by signing an informed consent.

#### 2.1.2. AD and MS Patients

A total of 46 patients, divided into two groups, were enrolled in a retrospective study at the Neurology Clinic of the University Hospital “Ospedali Riuniti” of Ancona and at the Neurology Unit of the “Madonna del Soccorso” Hospital of San Benedetto del Tronto, Italy. Each patient or caregiver signed informed consent before being enrolled. The first group, the AD group, comprised 19 patients diagnosed with probable Alzheimer’s disease according to 2011 McKhann criteria [29]. In their diagnostic work-up, they underwent brain imaging as well as a complete blood chemistry and metabolic (glucose, thyroid function and B vitamin status) panel to rule out secondary and potentially correctible causes of Cognitive Impairment. In no case were CSF pleocytosis or very elevated protein content (>80 mg/dL) found. Amyloid and Tau deposition markers were available on 15/19 patients.

The second group comprised 27 patients with Multiple Sclerosis diagnosed according to either the 2010 or 2017 modified McDonalds Criteria [30,31]. Each patient underwent brain and spinal cord imaging before CSF analysis. A total of 6/27 patients were given short courses of low-dose steroid therapy (cumulative doses inferior to 1000 mg prednisone equivalents) in the 4 months preceding the lumbar puncture. No patient in the MS group had history of Chronic or Episodic Migraines as ascertained from their medical record. The patient demographic characteristics are summarized in Table 1.

### 2.2. Lumbar Puncture

CSF, in AD and MS patients, was obtained in lateral decubitus from the L3–L4 interspace with a 20 G needle after an overnight fast. A total volume of 5–6 mL of CSF was obtained for each patient in order to carry out the routine analyses required for their clinical work-up. Aliquots contaminated with blood traces were discarded. Residual aliquots were stored at −20 °C until biochemical analyses. In Control patients, CSF was harvested on the day of the surgical procedure after an overnight fast in lateral decubitus at the L3–L4 interspace immediately before anesthetic administration. Aliquots contaminated with blood traces were discarded. A total volume of 3–4 mL was collected using a 20 G needle and immediately frozen at −20 °C until needed for biochemical analyses.

### 2.3. CSF Biochemical Analyses

Each patient underwent a physical–chemical CSF analysis. Patients in the AD group underwent Abeta 1-2, total Tau and p181-Tau measurement through commercially available ELISA Assays (INNOTEST Beta Amyloid 1-42, H-TAU, PHOSPHOTAU 181P, Fujirebio, JP), which were used according to the manufacturers’ instructions. CSF specimens from the MS group were subjected to a nephelometric panel, comprising oligoclonal bands, CSF albumin, CSF immunoglobulins and the Albumin quotient (Qalb).

### 2.4. α-CGRP Assay

The CSF α-CGRP content was determined through a commercially available ELISA assay (BioVendor—Laboratorni medicina a.s., Brno, Czech Republic), used according to the manufacturer’s protocol. The final absorbance was registered at 405–414 nm using a microplate reader (Synergy™ HTX, BioTek, Bernareggio, Italy), and the concentrations were subsequently interpolated from a standard curve and expressed as pg/mL.

Frozen CSF aliquots were thawed before being used in the analysis.

### 2.5. Neuropsychology

Patients in the AD group were administered a neuropsychological battery comprising tests exploring general cognition as well as specific cognitive domains.

#### 2.5.1. General Cognitive Status

The general cognitive status was explored by means of the Mini Mental State Examination (MMSE) [32].

#### 2.5.2. Memory

Short and long-term memories were explored. Both verbal and spatial components of working memory were explored by means of Digit Span and Corsi Blocks. Verbal long-term memory was investigated using the Rey AVLT and non-verbal long-term memory by means of the Rey–Osterrieth Figure B Test [33]. 

#### 2.5.3. Executive Functions and Attention

Executive functions and attention were studied by means of tests exploring single components of executive functions: motor planning was evaluated by means of Luria’s Motor Sequences, logical reasoning by means of Raven’s Progressive Matrices [34,35], and the Stroop test was used to measure selective attention.

#### 2.5.4. Language

Naming, reading and word comprehension were investigated by a locally-developed battery composed of a series of 40 items (10 figures of animals, 10 of clothes, 10 of fruits and 10 of tools). Comprehension was measured by a four-choice word–picture matching task. In addition, phonological fluency (FAS) and category fluency (three categories of knowledge, such as animals, colors and fruits) were explored.

#### 2.5.5. Ideomotor Praxis

Ideomotor Praxis was investigated by means of a standardized test. Patients were asked to perform a series of 20 gestures on both verbal command and imitation. Both upper limbs were tested [36]. 

#### 2.5.6. Visuoperception and Visuospatial Abilities

Visuoperception was investigated by means of Laila–Ghent Overlapping figures [37]. Each patient was shown two sets of overlapping figures and was asked to name the figure that they were able to identify. Visuospatial abilities were investigated by means of the copy of the Rey–Osterrieth Figure B Test, a complex geometrical figure.

#### 2.5.7. Neuroimaging

Patients in the MS group underwent high field (3T) brain and spinal cord MRI scans, and lesions were counted according to criteria expressed by Filippi et al. [38]. Patients in the AD group underwent brain imaging either by CT or MRI scans to exclude gross vascular or space-occupying lesions. For a single patient in the MS group, spinal cord imaging was not available at the time of the lumbar puncture.

### 2.6. Statistical Analyses

The Shapiro–Wilk test was used to assess the normality of quantitative variables. Variables showing an approximately normal distribution were described by the mean and standard deviation, and their means were confronted using parametrical analyses (one-way ANOVA with Tukey’s post-Hoc test). The relationship between quantitative variables showing an approximately normal distribution was assessed through correlation analyses (Pearson’s correlation coefficient) and linear models (linear regression and ANCOVA). 

As for neuropsychological testing, the relationship between test scores and the variables of interest was assessed calculating Spearman’s Rho Coefficients. In order to reduce testing on redundant predictors, so as to avoid influence from skewness or floor/ceiling effects in performance, only seven cognitive variables were included in the final analyses (a test representative of each cognitive domain was selected). Statistical analyses were performed with SPSS 20, Wolfram Mathematica 12 and R for Windows (version 4.1.2). Differences were considered significant at *p* < 0.05.

## 3. Results

### 3.1. Patient Characteristics

Table 1 shows the main demographic characteristics of the study population, as well as measures of disability and lesion burden for the MS group. Table 2 reports the main CSF parameters in the study groups. In the AD group, the mean Abeta 1–42 concentration was 613.93 pg/mL. According to a recent report [39], a concentration of Abeta 1–42 < 803 pg/mL does not contrast with the diagnosis of AD. The mean total and phosphorylated tau concentrations were, respectively, 580.27 and 69.93 pg/mL. According to Babapour and colleagues, a total tau concentration >374 pg/mL could be considered suggestive of neurodegeneration [39]. As for general cognitive measures, the mean MMSE score was 19.73, which denotes a condition of moderate dementia.

As for the MS group, Oligoclonal bands were detected in 25/27 patients, while very elevated Qalb (>9), was detected in 4/27 patients. The median EDSS score was 1.0, representing patients with minimal disability. 

### 3.2. α-CGRP CSF Content in AD, MS and Control Subjects; Relationship with Protein Content

**Figure 1:**
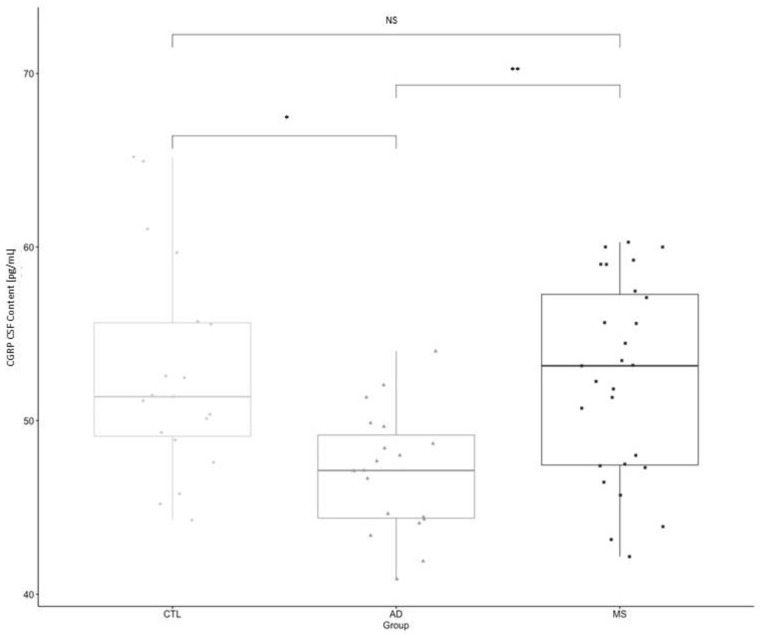
Comparison of the mean CSF concentration of α-CGRP between Control Subjects, Alzheimer Disease and Multiple Sclerosis. Legend: *: *p* = 0.008 from One-Way ANOVA (Alzheimer’s Disease vs. Controls); **: *p* = 0.004 from One-Way ANOVA (Alzheimer’s Disease vs. Multiple Sclerosis).

The mean α-CGRP measured concentration in the AD group was 47.06 (±3.5) pg/mL; in the MS group, was 52.26 (±5.5) pg/mL; and in control subjects, was 52.05 (±5.7) pg/mL (Table 2). One-way ANOVA with post-Hoc comparisons (F = 7.2096; *p* < 0.01) showed a statistically significant difference between AD and Control Subjects, as well as between MS and AD patients (*p* = 0.004—AD vs. MS; *p* = 0.008—AD vs. Controls; Figure 1). No difference was found between control subjects and MS patients (*p* = 0.97). 

Overall, the protein content was not significantly different between the AD and MS groups (Protein_AD_ = 43.7 ± 16.15 mg/dL; Protein_MS_ = 38.75 ± 11.4 mg/dL; *p* = 0.25). In a subsequent ANCOVA model with age, gender and group as covariates, neither Gender (F = 1.48; *p* = 0.74), nor Age (F = 0.45, *p* = 0.50) accounted for the observed difference (F_age_ = 1.721; *p* = 0.198; F_group_ = 5.293; *p* = 0.008). Subsequently, a correlation analysis between the CGRP and protein content was performed in both groups. In MS and AD patients, no relationship was observed (rMS = 0.14; *p* = 0.47; rAD = −0.37; *p* = 0.12). 

### 3.3. CSF α-CGRP: Relationship with Amyloid, Tau Pathology Markers and Scores on Neuropsychological Testing

In AD patients, we did not observe a significant association between α-CGRP and Amyloid Beta 1–42 (rhoabeta = −0.100; *p*= 0.723), Total Tau (rhotau = 0.251; *p* = 0.367) or Phosphorylated Tau (rhoptau = 0.221; *p*= 0.428). In cognitive testing, no association was found between α-CGRP and measures of general cognition, such as the MMSE. Analyzing distinct neuropsychological domains, an association between the α-CGRP concentration and performance in selective attention and visuoperceptive functions was observed.

We found a statistically significant moderate negative relationship with Stroop Test Type II corrections (rho = −0.577; *p* = 0.039); while in visuoperceptive functions, a statistically significant moderate positive relationship between α-CGRP and Laila–Ghent figure recognition scores was found (rho = 0.586; *p* = 0.028). Regarding attention and executive functions, only Type II Stroop error and correction numbers were selected for correlation analyses since the Type II test is thought to be more representative of Selective Attention given the greater magnitude of interference. The mean scores for all neuropsychological tests and correlation coefficients for all the analyzed variables are reported in Table 3.

### 3.4. CSF α-CGRP: Relation to Nephelometric Indexes and Lesion Burden in Multiple Sclerosis

The relationship between the α-CGRP CSF content and intrathecal inflammatory markers was further examined assessing whether α-CGRP concentration was related to the IgG content. No correlation was found (r = 0.06; *p* = 0.97). The α-CGRP CSF content did not appear to be significantly influenced by blood brain barrier permeability, as it appeared to relate poorly to the CSF albumin quotient (r = 0.19; *p* = 0.31). 

**Figure 2:**
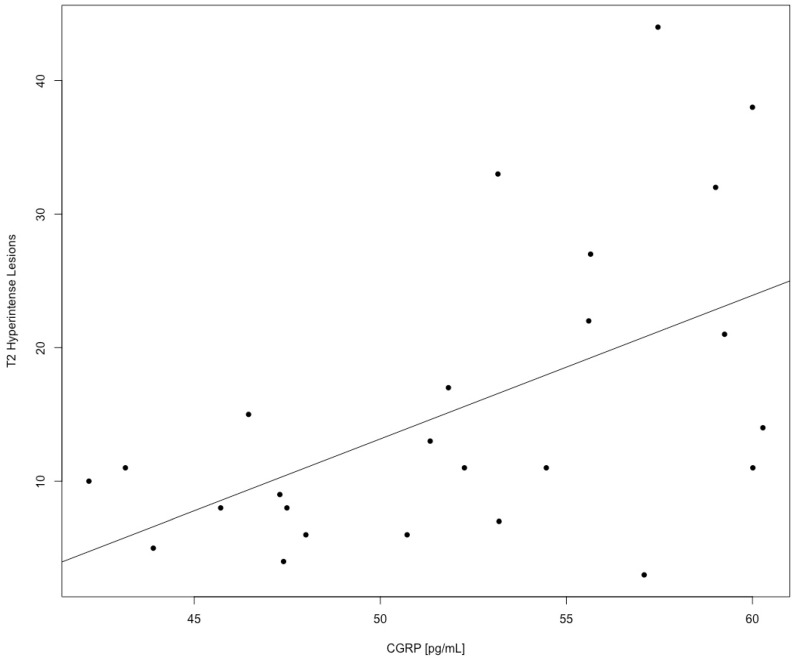
Linear regression plot of the α-CGRP CSF Concentration vs. Total T2 Hyperintense lesion count on MRI (*p* = 0.005, Multiple R squared 0.29—Adjusted R-Squared 0.27).

As for the disease phenotype in Multiple Sclerosis, a positive, statistically significant association between T2 hyperintense lesion count and higher α-CGRP concentration (r = 0.54; *p* = 0.005, Adjusted R-Squared = 0.27; Figure 2) was observed as a raw correlation and in a univariate linear regression model, while no significant relationship between the α-CGRP CSF content and either the T2 positive spinal cord lesion count (r = 0.11, *p* = 0.75) or total contrast-enhancing lesion number (r = −0.06, *p* = 0.75) was found.

## 4. Discussion

In the present study, we compared the cerebrospinal fluid α-CGRP concentration between Multiple Sclerosis, Alzheimer’s disease and control subjects, finding equal levels in MS in comparison to the controls, while also finding, as shown in a previous study, a lower α-CGRP level in dementia patients in comparison to the control subjects. α-CGRP has been regarded as a possible marker of dementia and/or depression; however, it has never been heretofore compared to specific neuropsychological profiles and/or to AD core marker status [19]. 

Our results could represent anecdotal evidence supporting the hypothesis of an association between a lower α-CGRP concentration and a worse neuropsychological profile, albeit in specific domains, selective attention and visuoperceptual functions. A reduction in its concentration should be cautiously interpreted as it might reflect the status of multiple biological pathways connected to its synthesis (by both neural and non-neural cells) as well as clearance through different processes (i.e., neuronal reuptake, enzymatic degradation by IDE and Neprilysin [12] and glymphatic outflow) and a single measurement might not be sufficient to infer mechanistic pathogenetic insights. 

However, in the AD group, the absence of an association between the CSF protein content and α-CGRP concentration could support the hypothesis that its level might reflect phenomena occurring within the CNS, perhaps deriving from upstream degenerative processes. If its anatomical expression is considered, as well as its emerging role in sustaining hippocampal plasticity, we could have expected to observe an association between α-CGRP levels and memory performances, although the small sample size and selection of patients affected by mild or more severe dementia, characterized by severe memory impairment at the time of examination might have reduced the chances to detect an association. 

Several reports showed the appearance of Tau pathology in the brainstem of Alzheimer disease patients [40], as well as in the parabrachial nucleus, starting from very early neuropathological stages [41]. A potential association with visuoperceptual functions, however, might be speculatively linked on an anatomical basis to CGRPergic circuits connecting subcortical structures (such as the parabrachial nucleus and/or the amygdala) with temporal lobe associative cortices, which are commonly affected in AD [42] and warrant further investigation. Furthermore, α-CGRP has been recently linked to high affinity activation of amylin type 1α receptors, and amylin is thought to play a protective role in AD [43,44]. α-CGRP has also been related to nicotinic receptor activity modulation, in both peripheral and central scenarios [45]. 

Since specific nicotinic receptor subtypes (i.e., α7nAchR) are involved in both central cholinergic transmission, neurotransmitter release [46] as well as immune processes [47], this could speculatively constitute a possible common pathway in AD and MS. In our cohort, we did not detect an association between amyloid and tau deposition markers and α-CGRP concentration. These results need to be confirmed on wider cohorts and in different disease stages, although the aforementioned markers can be interpreted as state markers, and thus they might not vary according to clinical severity over the course of the disease [10]. 

As for methodological aspects, in comparison to previous studies on the same subject, we used an ELISA assay for α-CGRP measurements, instead of RIA. The higher sensibility of this method might therefore explain the higher measurements obtained. In comparison to the most recent paper on this subject, the study from Svenningson and colleagues, we obtained comparable measurements notwithstanding the differences in storage conditions and analytical methods [48]. Furthermore, as previously described, the α-CGRP concentration appeared to not be significantly influenced by age and sex differences [25,26].

As for Multiple Sclerosis, we observed a positive association with the demyelinating lesion burden, measured as the total lesion count, independently from the Qalb values, which represent the entity of blood brain barrier leakage and, therefore, speculatively reflecting intrathecal dynamics, rather than CGRP plasma to CSF transfer. We did not detect an association with the active, contrast-enhancing or spinal cord lesion burden, which might have been influenced by the sample size, but also by the selection of patients mostly at the beginning of their disease course, with a predominantly supratentorial lesion load and a relapsing remitting course.

On the whole, the data presented here suggest a possible association between a higher CGRP and the extension of demyelinating lesions, perhaps pinpointing an involvement for CGRP in demyelination processes. We are wary that our study possesses some limits; firstly, its retrospective nature, which does not allow to draw conclusions about cause–effect relationships; secondarily, for Multiple Sclerosis, no CSF markers of axonal loss or of immunophenotype were measured, thus, not allowing us to assess a relationship between CGRP content and neuroaxonal loss or specific inflammatory immunophenotypes. 

Given the complexity of demyelinating lesions under a pathophysiological perspective [49] and the coexistence in each individual, even from early disease phases, of lesions with heterogeneous degrees of inflammatory activity and neuroaxonal loss independently from contrast-enhancement, no firm conclusion, under a pathophysiological perspective, can be drawn about the association between CGRP CSF content, CNS acute or chronic inflammation and disease activity.

Under a theoretical perspective, in line with the described pleiotropic immunomodulatory and neuroprotective properties, as well as its expression in spinal motor neurons and the ameliorative effects described in EAE models, the data here presented might support the hypothesis that CGRP, and related peptides could be involved in Multiple Sclerosis pathogenesis; therefore, the relationship between α-CGRP CSF content and lesion burden might be of interest for further evaluation.

## Figures and Tables

**Table 1:** Summary of the demographic and MRI characteristics of the study cohort.

	Control Subjects (*n* = 17)	Alzheimer’s Disease (*n* = 19)	Multiple Sclerosis (*n* = 27)
Variable	Mean (SD)	Mean (SD)	Mean (SD)
Age	56.5 (±18)	72.7 (±7.2)	40.3 (±14.4)
Gender (M/F)	10/17	8/11	7/20
Age at Onset (years)	n.a.	69.8 (±6.8)	39.3 (±13)
Duration of Disease (years)	n.a.	2.8 (±1.7)	1.9 (±2.4)
Education (years)	n.a.	8.4 (±4.5)	n.a.
EDSS	n.a.	n.a.	1.0 *
Total T2 Hyperintensities	n.a.	n.a.	11 (7−18) *
Spinal cord T2 Hyperintensities	n.a.	n.a.	2 (1−4) *
Patients with spinal cord lesions	n.a.	n.a.	21/27
Patients with contrast-enhancing lesions	n.a.	n.a.	17/27

The results are expressed as the mean ± SD. Legend: n.a.: not available. *: results expressed as the median and interquartile range.

**Table 2:** Overview of the CSF physical–chemical, nephelometric parameters and α-CGRP in the study cohort.

	Control Subjects (*n* = 17) Mean (±SD)	Alzheimer’s Disease (*n* = 19) Mean (±SD)	Multiple Sclerosis (*n* = 27) Mean (±SD)	*p*
α-CGRP (pg/mL)	52.05 (±5.7)	47.06 (±3.5)	52.26 (±5.5)	#
CSF protein content (mg/dl)	n.a.	43.7 (±16.15)	38.75 (±11.4)	0.25
Abeta 1–42 (pg/mL) *	n.a.	613.93 (±171.58)	n.a.	n.a.
Total Tau (pg/mL) *	n.a.	580.27 (±376.17)	n.a.	n.a.
P-Tau181 (pg/mL) *	n.a.	69.93 (±49.9)	n.a.	n.a.
CSF albumin	n.a.	n.a.	22.3 (±10.20)	n.a.
CSF IgG	n.a.	n.a.	4 (±2)	n.a.
Q_alb_	n.a.	n.a.	6.46 (±6.57)	n.a.
Link Index	n.a.	n.a.	0.83 (±0.42)	n.a.
Oligoclonal Bands	n.a.	n.a.	25/27	n.a.

Legend: #: *p* = 0.004 for AD vs. MS; *p* = 0.008 for Controls vs. AD; *p* = 0.97 for Controls vs. MS (One-Way ANOVA with Tukey’s Post-Hoc test). *: (available on 15 patients on 19); n.a.: not available.

**Table 3:** Scores in general and domain-specific neuropsychological tests in the AD cohort. Relationship between CSF α-CGRP content and neuropsychological performance in selected domain-specific tests. Scoring is expressed as the mean and standard deviation.

Test	Mean (SD)	Spearman Rho	*p*
*Global mental status*			
MMSE	19.73 (± 4.45)	0.196	n.s.
*Executive functions and attention*			
Raven’s Progressive Matrices	17.3 (±7.6)	n.a.	n.a.
Luria Motor Sequences	30.60(±17.78)	n.a.	n.a.
Stroop Test I (time)	48.8 (±17.6)	n.a.	n.a.
Stroop Test II (time)	102.84 (± 38.6)	n.a.	n.a.
Stroop Test I (errors)	0.5 (±1.1)	n.a.	n.a.
Stroop Test II (errors)	2.69 (±4.1)	−0.191	n.s.
Stroop I (corrections)	0.29 (±0.61)	n.a.	n.a.
Stroop II (corrections)	1.77 (±3.4)	−0.577	0.039
*Memory*			
Digit Span	4.57 (±0.94)	n.a.	n.a.
Corsi Cubes	3.79 (±0.8)	n.a.	n.a.
Short Term type B Rey figure	8.36 (±5.27)	n.a.	n.a.
Long Term type B Rey figure	5.47 (±5.68)	n.a.	n.a.
AVLT Total Effect	18.8 (±7.24)	n.a.	n.a.
AVLT Long Term	0.5714 (±0.75)	n.a.	n.a.
*Visuoperceptual and visuospatial functions*			
LG Overlapping Figures	8.57 (±0.75)	0.586	0.028
Type B Rey Figure Copy	22.84 (±8.5)	n.a.	n.a.
*Praxis*			
Ideomotor Praxis Dominant Hand	19.75 (±1)	0.140	n.s.
Ideomotor Praxis Nondominant Hand	19.63 (±1.1)	n.a.	n.a.
*Language*			
Semantic Fluency	25.2 (±10)	n.a.	n.s.
Phonemic Fluency	20.47 (±10.77)	−0.281	n.s.
Naming	30.6 (±12.2)	n.a.	n.a.
Reading	40 (±0)	n.a.	n.a.
Comprehension	39.83 (±0.41)	n.a.	n.a.

Legend: n.a.: not available; n.s.: not significant. AVLT: Rey Auditory Verbal Learning Test.

## Data Availability

Not applicable.

## References

[1] WHO (2017). Global Action Plan on the Public Health Response to Dementia 2017–2025.

[2] De Strooper B., Karran E. (2016). The Cellular Phase of Alzheimer’s Disease. Cell.

[3] Daborg J., Andreasson U., Pekna M., Lautner R., Hanse E., Minthon L., Blennow K., Hansson O., Zetterberg H. (2012). Cerebrospinal fluid levels of complement proteins C3, C4 and CR1 in Alzheimer’s disease. J. Neural Transm..

[4] Bergamaschini L., Parnetti L., Pareyson D., Canziani S., Cugno M., Agostoni A. (1998). Activation of the Contact System in Cerebrospinal Fluid of Patients with Alzheimer Disease. Alzheimer Dis. Assoc. Disord..

[5] Baecher-Allan C., Kaskow B.J., Weiner H.L. (2018). Multiple Sclerosis: Mechanisms and Immunotherapy. Neuron.

[6] Ontaneda D., Thompson A.J., Fox R.J., A Cohen J. (2016). Progressive multiple sclerosis: Prospects for disease therapy, repair, and restoration of function. Lancet.

[7] Kawachi I., Lassmann H. (2016). Neurodegeneration in multiple sclerosis and neuromyelitis optica. J. Neurol. Neurosurg. Psychiatry.

[8] Pietroboni A.M., Colombi A., Carandini T., Scarpini E., Galimberti D., Bozzali M. (2020). The Role of Amyloid-β in White Matter Damage: Possible Common Pathogenetic Mechanisms in Neurodegenerative and Demyelinating Diseases. J. Alzheimer’s Dis..

[9] Stenager E. (2019). A global perspective on the burden of multiple sclerosis. Lancet Neurol..

[10] Parnetti L., Chiasserini D., Eusebi P., Giannandrea D., Bellomo G., De Carlo C., Padiglioni C., Mastrocola S., Lisetti V., Calabresi P. (2012). Performance of Aβ1-40, Aβ1-42, Total Tau, and Phosphorylated Tau as Predictors of Dementia in a Cohort of Patients with Mild Cognitive Impairment. J. Alzheimer’s Dis..

[11] Paul A., Comabella M., Gandhi R. (2018). Biomarkers in Multiple Sclerosis. Cold Spring Harb. Perspect. Med..

[12] Russell F.A., King R., Smillie S.-J., Kodji X., Brain S.D. (2014). Calcitonin Gene-Related Peptide: Physiology and Pathophysiology. Physiol. Rev..

[13] Charles A. (2018). The pathophysiology of migraine: Implications for clinical management. Lancet Neurol..

[14] Warfvinge K., Edvinsson L., Pickering D.S., Sheykhzade M. (2019). The Presence of Calcitonin Gene-Related Peptide and Its Receptors in Rat, Pig and Human Brain: Species Differences in Calcitonin Gene-Related Peptide Pharmacology. Pharmacology.

[15] Arvidsson U., Piehl F., Johnson H., Ulfhake B., Cullheim S., Hökfelt T. (1993). The peptidergic motoneurone. Neuroreport.

[16] Mathé A.A., Hertel P., Nomikos G.G., Gruber S., Mathé J.M., Svensson T.H. (1996). The psychotomimetic drugs d-amphetamine and phencyclidine release calcitonin gene-related peptide in the limbic forebrain of the rat. J. Neurosci Res..

[17] Kovács A., Telegdy G. (1995). Effects of CGRP on active avoidance behavior in rats. Physiol. Behav..

[18] Borkum J.M. (2019). CGRP and Brain Functioning: Cautions for Migraine Treatment. Headache: J. Head Face Pain.

[19] Angelucci F., A Ellenbroek B., El Khoury A., Mathé A.A. (2018). CGRP in a gene–environment interaction model for depression: Effects of antidepressant treatment. Acta Neuropsychiatr..

[20] Hashikawa-Hobara N., Ogawa T., Sakamoto Y., Matsuo Y., Ogawa M., Zamami Y., Hashikawa N. (2015). Calcitonin gene-related peptide pre-administration acts as a novel antidepressant in stressed mice. Sci. Rep..

[21] Harzenetter M.D., Novotny A.R., Gais P., Molina C.A., Altmayr F., Holzmann B. (2007). Negative Regulation of TLR Responses by the Neuropeptide CGRP Is Mediated by the Transcriptional Repressor ICER. J. Immunol..

[22] Giardino L., Giuliani A., Fernandez M., Calzà L. (2004). Spinal motoneurone distress during experimental allergic encephalomyelitis. Neuropathol. Appl. Neurobiol..

[23] Rossetti I., Zambusi L., Finardi A., Bodini A., Provini L., Furlan R., Morara S. (2018). Calcitonin gene-related peptide decreases IL-1beta, IL-6 as well as Ym1, Arg1, CD163 expression in a brain tissue context-dependent manner while ameliorating experimental autoimmune encephalomyelitis. J. Neuroimmunol..

[24] Zhai L., Sakurai T., Kamiyoshi A., Ichikawa-Shindo Y., Kawate H., Tanaka M., Xian X., Hirabayashi K., Dai K., Cui N. (2018). Endogenous calcitonin gene-related peptide suppresses ischemic brain injuries and progression of cognitive decline. J. Hypertens..

[25] Mathé A.A., Agren H., Wallin A., Blennow K. (2002). Calcitonin gene-related peptide and calcitonin in the CSF of patients with dementia and depression: Possible disease markers. Prog. Neuro-Psychopharmacol. Biol. Psychiatry.

[26] Mathé A.A., Ågren H., Lindström L., Theodorsson E. (1994). Increased concentration of calcitonin gene-related peptide in cere-brospinal fluid of depressed patients. A possible trait marker of major depressive disorder. Neurosci. Lett..

[27] Wimalawansa S., MacIntyre I. (1987). The Presence of Calcitonin Gene-Related Peptide in Human Cerebrospinal Fluid. Brain.

[28] Na H., Gan Q., Mcparland L., Yang J., Yao H., Tian H., Zhang Z., Qiu W.Q. (2020). Characterization of the effects of calcitonin gene-related peptide receptor antagonist for Alzheimer’s disease. Neuropharmacology.

[29] McKhann G.M., Knopman D.S., Chertkow H., Hyman B.T., Jack C.R., Kawas C.H., Klunk W.E., Koroshetz W.J., Manly J.J., Mayeux R. (2011). The diagnosis of dementia due to Alzheimer’s disease: Recommendations from the National Institute on Aging-Alzheimer’s association workgroups on diagnostic guidelines for Alzheimer’s disease. Alzheimers Dement. J. Alzheimers Assoc..

[30] Polman C.H., Reingold S.C., Banwell B., Clanet M., Cohen J.A., Filippi M., Fujihara K., Havrdova E., Hutchinson M., Kappos L. (2011). Diagnostic criteria for multiple sclerosis: 2010 Revisions to the McDonald criteria. Ann. Neurol..

[31] Thompson A.J., Banwell B.L., Barkhof F., Carroll W.M., Coetzee T., Comi G., Correale J., Fazekas F., Filippi M., Freedman M.S. (2018). Diagnosis of multiple sclerosis: 2017 revisions of the McDonald criteria. Lancet Neurol..

[32] Folstein M.F., Folstein S.E., McHugh P.R. (1975). “Mini-mental state”. A practical method for grading the cognitive state of pa-tients for the clinician. J. Psychiatr Res..

[33] Luzzi S., Pesallaccia M., Fabi K., Muti M., Viticchi G., Provinciali L., Piccirilli M. (2011). Non-verbal memory measured by Rey–Osterrieth Complex Figure B: Normative data. Neurol. Sci..

[34] Raven J. (1958). Guide to Using the Coloured Progressive Matrices.

[35] Caltagirone C., Gainotti G., Masullo C., Miceli G. (1979). Validity of some neuropsychological tests in the assessment of mental de-terioration. Acta Psychiatr Scand..

[36] (1987). Italian standardization and classification of Neuropsychological tests. The Italian Group on the Neuropsychological Study of Aging. Ital. J. Neurol Sci..

[37] Sala S.D., Laiacona M., Trivelli C., Spinnler H. (1995). Poppelreuter-Ghent’s overlapping figures test: Its sensitivity to age, and its clinical use. Arch. Clin. Neuropsychol..

[38] Filippi M., Preziosa P., Banwell B.L., Barkhof F., Ciccarelli O., De Stefano N., Geurts J.J.G., Paul F., Reich D.S., Toosy A.T. (2019). Assessment of lesions on magnetic resonance imaging in multiple sclerosis: Practical guidelines. Brain.

[39] Mofrad R.B., Schoonenboom N.S., Tijms B.M., Scheltens P., Visser P.J., Van Der Flier W.M., Teunissen C.E. (2018). Decision tree supports the interpretation of CSF biomarkers in Alzheimer’s disease. Alzheimer’s Dementia: Diagn. Assess. Dis. Monit..

[40] Parvizi J., Van Hoesen G.W., Damasio A. (1998). Severe pathological changes of parabrachial nucleus in Alzheimerʼs disease. NeuroReport.

[41] Rueb U., Stratmann K., Heinsen H., Del Turco D., Seidel K., Dunnen W.D., Korf H. (2016). The Brainstem Tau Cytoskeletal Pathology of Alzheimer’s Disease: A Brief Historical Overview and Description of its Anatomical Distribution Pattern, Evolutional Features, Pathogenetic and Clinical Relevance. Curr. Alzheimer Res..

[42] Jagust W.J., Eberling J.L., Richardson B.C., Reed B.R., Baker M.G., Nordahl T.E., Budinger T.F. (1993). The cortical topography of temporal lobe hypometabolism in early Alz-heimer’s disease. Brain Res..

[43] Hay D.L., Garelja M.L., Poyner D.R., Walker C.S. (2017). Update on the pharmacology of calcitonin/CGRP family of peptides: IUPHAR Review 25. J. Cereb. Blood Flow Metab..

[44] Wang E., Zhu H., Wang X., Gower A.C., Wallack M., Blusztajn J.K., Kowall N., Qiu W.Q. (2017). Amylin Treatment Reduces Neuroinflammation and Ameliorates Abnormal Patterns of Gene Expression in the Cerebral Cortex of an Alzheimer’s Disease Mouse Model. J. Alzheimer’s Dis..

[45] Di Angelantonio S., Giniatullin R., Costa V., Sokolova E., Nistri A. (2003). Modulation of neuronal nicotinic receptor function by the neuropeptides CGRP and substance P on autonomic nerve cells. Br. J. Pharmacol..

[46] De Jaco A., Bernardini L., Rosati J., Tata A.M. (2017). Alpha-7 Nicotinic Receptors in Nervous System Disorders: From Function to Therapeutic Perspectives. Central Nerv. Syst. Agents Med. Chem..

[47] Siniavin A.E., Streltsova M.A., Kudryavtsev D.S., Shelukhina I.V., Utkin Y.N., Tsetlin V.I. (2020). Activation of α7 Nicotinic Acetylcholine Receptor Upregulates HLA-DR and Macrophage Receptors: Potential Role in Adaptive Immunity and in Preventing Immunosuppression. Biomolecules.

[48] Svenningsson P., Pålhagen S., Mathé A.A. (2017). Neuropeptide Y and Calcitonin Gene-Related Peptide in Cerebrospinal Fluid in Parkinson’s Disease with Comorbid Depression versus Patients with Major Depressive Disorder. Front. Psychiatry.

[49] Lassmann H. (2018). Multiple Sclerosis Pathology. Cold Spring Harb. Perspect. Med..

